# User Involvement in Transition Care in Virtual 4‐Party Meetings: A Qualitative Study

**DOI:** 10.1111/hex.70566

**Published:** 2026-01-24

**Authors:** Ditte Høgsgaard, Janet Froulund Jensen, Heidi Myglegård Andersen

**Affiliations:** ^1^ Department of Regional Health Research University of Southern Denmark Odense Denmark; ^2^ Research and Implementation Unit PROgrez, Naestved Slagelse and Ringsted Hospital Slagelse Denmark; ^3^ Primary and e‐Health Care, Region Zealand Soroe Denmark; ^4^ Department of Neurologi Sjaellands University Hospital Roskilde Denmark

**Keywords:** integrated care, multimorbidity, transitional care model, user involvement, video consultation

## Abstract

**Background:**

Older adults with multimorbidity often experience fragmented transitional care between the hospital, primary care, and municipal services. Insufficient communication and limited user involvement can compromise safety, satisfaction, and continuity. To address these challenges, a structured cross‐sectoral intervention, the Virtual 4‐Party Meeting (V4M), was developed to enhance patient and family involvement in discharge planning.

**Aim:**

To explore how older adults with multimorbidity and their relatives experienced involvement in transitional care through V4M.

**Methods:**

A qualitative hermeneutic design was applied. Eleven patients ( + 65 years) and their relatives participated in semi‐structured interviews immediately after V4M and again 14 days post‐discharge. Data were analyzed using Braun and Clarke's reflexive thematic analysis within a Gadamerian hermeneutic framework.

**Results:**

Three themes emerged: (1) Bridges between Systems. V4M reduced fragmentation and improved coordination through shared dialog; (2) A Relational Space of Alignment, the meetings created emotional safety and supported patient autonomy and relational understanding; and (3) Involvement and Responsibility are deeply interconnected. Meaningful involvement occurred when accountability was shared between patients, relatives, and professionals.

**Conclusion:**

V4M provided an effective model for integrating user involvement into transitional care by combining structural coordination with relational engagement. Patients and relatives felt acknowledged, informed, and reassured when professionals gained a clearer sense of shared responsibility. The study highlights that genuine user involvement depends on both emotional recognition and concrete accountability mechanisms across sectors.

**Patient or Public Contribution:**

Older adults with multimorbidity and their relatives contributed to the development of the V4M intervention. In this study, patients and relatives participated as interviewees but were not involved in data analysis or manuscript preparation.

## 1 Introduction

The increasing number of older adults with multimorbidity poses significant challenges to healthcare systems worldwide [1, 2, 3]. These individuals frequently experience hospitalizations and complex transitions between healthcare sectors, making effective coordination essential [4, 5]. Transitional care, especially for frail older adults, is complicated by fragmented services, inconsistent communication, and unclear role responsibilities among professionals across various settings [6, 7, 8, 9]. Research indicates that older patients and their families are often inadequately involved in discharge planning and follow‐up care [10, 11].

Ineffective communication and information transfer during care transitions can significantly impact patient safety and continuity of care [12, 13, 14]. This lack of communication undermines continuity of care, patient safety, and satisfaction. Moreover, studies have shown that when patients are involved in the planning process, care can be tailored to their needs, preferences, and values [15, 16]. A qualitative study showed that older people frequently experience limited support when expressing their preferences, highlighting the need for tailored decision‐making approaches in transitional care [17]. Similarly, van Grootel et al. [18] demonstrated that when patients actively contribute to the design of transitional pathways, it fosters more responsive and effective care coordination. For instance, Hansen et al. [19] showed that nurses may struggle to incorporate patients' perspectives in electronic health records covering discharge planning. Petersen et al. [20] observed that involving frail older adults with complex needs is challenging. They noted that unfamiliarity among health professionals and unclear responsibilities hinder patient involvement, recommending closer cooperation and joint planning to enhance engagement. Beck et al. [21] have shown that a lack of participation of older adults significantly impacts transitional care and the follow‐up process. If the patient is not involved in transitioning from the hospital to follow‐up care in primary care, it creates unsafe and dangerous situations and an unclear perception of the roles and responsibilities of patients, families, and healthcare professionals. It is also well known that the health care professionals are more likely to reflect their values and preferences when patients and relatives are actively involved in planning and decisions [22]. By sharing their opinions and experiences, these users communicate their concerns and expectations directly to service providers, promoting a better understanding and responsiveness.

Additionally, they take an active role in decision‐making processes, becoming partners in the co‐production of their care and contributing to the development of tailored health services [22]. This approach recognizes that user involvement can significantly enhance transitional care, in which patient engagement is vital for successful outcomes. Specifically, it may improve continuity and coordination of care across sectors [2], patient and family satisfaction with the discharge process, patients' perceived self‐efficacy and ability to manage care at home, and the reduction of preventable readmissions. These dimensions reflect the potential impact of user participation at both the individual and system levels.

Although numerous studies [11, 23] have explored communication challenges during hospital‐to‐home transitions and interventions to improve coordination, limited attention has been paid to how older patients and their relatives experience active involvement in cross‐sectoral discharge planning. Existing research often focuses on professional collaboration or readmission outcomes, rather than on how user involvement is operationalized and experienced within structured, cross‐sectoral settings. The Virtual 4‐Party Meeting (V4M) [24, 25] represents an innovative approach that integrates hospital, primary care, and municipal actors with the patient and their relatives in a single structured dialog before discharge. However, empirical knowledge about how this format influences patients' and relatives' sense of involvement, shared responsibility, and continuity of care remains scarce. This study addresses the following knowledge gap: how do older adults with multimorbidity and their relatives experience involvement in transitional care when participation is facilitated through V4M?

### 1.1 Aim

This study used a qualitative hermeneutic approach to explore the experiences of older patients and their relatives in involvement in transitional care through V4M.

### 1.2 Methods

#### 1.2.1 Design

Hermeneutics is well‐suited for examining meaning‐making in health‐related experiences, emphasizing contextual understanding, interpretation, and the dynamic interaction between researcher and participants [26, 27]. The approach draws on Gadamer's philosophical hermeneutics, highlighting the role of pre‐understanding, dialog, and interpretation in achieving deeper insights [28].

#### 1.2.2 Intervention: Virtual 4‐Meetings (V4Ms)

V4M is a structured, 30‐min video‐based intervention designed to enhance user involvement and coordination in discharge planning for older adults with multimorbidity [24, 25]. The meeting brings together four key parties: (1) the patient and one or more relatives, (2) hospital professionals (a nurse and a physician), (3) a municipal nurse, and (4) the patient's general practitioner (GP).

The purpose of the V4M is to create a shared understanding of the patient's health status, priorities, and care needs before discharge, ensuring that all responsible parties agree on a coordinated follow‐up plan. The central question guides the conversation: “What is most important to you right now?” This encourages the patient to express concerns and preferences that inform goal‐setting and care planning [29]. Furthermore, health professionals can help by evaluating the need for transitional care and, for very ill patients, the requirement for terminal and spiritual support. V4M is scheduled to occur as soon as possible after hospital admission, usually on the third or fourth day. A designated hospital coordinator identifies eligible patients and contacts all participants to ensure that the meeting is arranged at a time suitable for both hospital staff and primary care partners. Meetings are held in the patient's hospital room, with the patient and relatives physically present. The GP and municipal nurse participate via a secure videoconference platform compliant with Danish data protection standards.

Each meeting lasts approximately 25–35 min and follows a standard agenda to ensure that all key domains are covered: (1) Patient's primary concerns and priorities; (2) Summary of hospital treatment and status; (3) Discussion of follow‐up care and allocation of responsibilities; and (4) Agreement on next steps and confirmation of contact persons [29].

The hospital nurse facilitates the session, ensuring that all voices are heard and that the meeting concludes with a clearly defined action plan. The videoconferences use an encrypted, hospital‐approved platform that allows real‐time audio and visual interaction. A large screen is positioned to ensure that all participants can see and communicate clearly, fostering an atmosphere of equality and inclusion. Each meeting ends with a written summary that confirms responsibilities across sectors. This summary is given to the patient and their family, shared digitally with all participants, and uploaded to the electronic health record to ensure transparency, continuity, and accountability.

### 1.3 Participants

V4M was conducted in two medical wards at a regional hospital in Region Zealand and two municipalities. Patients who were included in the interviews had participated in V4M. Patients had to be 65 years of age or older, have two or more chronic conditions, be able to understand and speak Danish, and provide informed consent. Exclusions included severe cognitive impairment, frailty, or language barriers. A designated hospital coordinator recruited participants. This recruitment strategy may have favored patients who were clinically stable, cognitively able, and willing to participate in a video‐based meeting. Relatives were included if identified by the patient and consented to participate. The study therefore applied a patient‐centered sampling approach, with optional inclusion of relatives, rather than a predefined dyad methodology.

### 1.4 Data Collection

The first author conducted qualitative interviews [30] in two rounds to capture both immediate reactions and more reflective experiences after returning home: the first round took place immediately after V4M at the hospital, and the second round approximately 14 days after discharge.

The rationale for this sample size is based on considerations: our analytical approach (Gadamer + Braun & Clarke) emphasizes iterative interpretation and a continual movement between part and whole. Thus, “sufficient” data are judged by whether the material has achieved hermeneutic sufficiency (information power) to develop stable themes, not by a formal sample size.

The combination of immediate (experienced) and ~14‐day reflective interviews made it possible to identify both short‐term reactions and more considered assessments of involvement and responsibility. This approach enhanced the credibility and reflexive depth of the analysis by capturing variation in meaning‐making over time. Some planned follow‐up interviews could not be conducted (e.g., due to deterioration in health or death), as reflected in. This affected the total number but not the hermeneutic judgment of sufficiency. The judgment that additional interviews yielded few new interpretive insights is documented in our audit trail (memos, decision log, and team discussions), ensuring transparency in the sample size choice.

In summary, 19 interviews represented a methodologically well‐founded choice: they provided sufficient information power for in‐depth hermeneutic interpretation within a heterogeneous, multimorbid participant group. An interview guide was used to focus on how the patient experience involves the discharge process (Supporting File A). Interviews lasted 15–40 min (mean 28). The interviews were recorded and transcribed.

The study adhered to COREQ guidelines [31] for transparency and rigor in reporting qualitative findings (Supporting File B).

### 1.5 Data Analysis

Gadamer's philosophical hermeneutics provided the overarching interpretive framework for this study, guiding our understanding of meaning, reflexivity, and dialog between researcher and participant [28]. Within this epistemological orientation, Braun and Clarke's six‐step approach to thematic analysis was applied as a systematic and transparent method for identifying, organizing, and interpreting themes [32].

**FIGURE 1:**
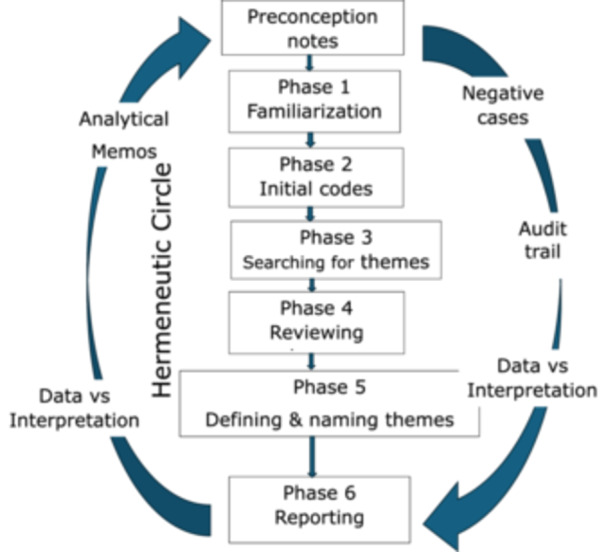
Combining Gadamer's hermeneutic circle and Braun & Clarke's reflexive thematic analysis.

Figure 1 illustrates how we combine Gadamer‐inspired hermeneutics and Braun & Clarke's reflexive thematic analysis. The data analysis process begins with documented preconceptions, follows Braun & Clarke's six phases, and integrates hermeneutic practices (the hermeneutic circle, analytical memos, peer debriefing, and an audit trail) that continuously ensure reflexivity, transparency, and in‐depth interpretation. Before coding, each researcher recorded their preconceptions and expectations, which were used actively throughout the analysis as part of the audit trail. Coding and theme development proceeded iteratively, moving back and forth several times between individual quotations (parts) and the overarching thematic understanding (whole) through an explicit use of the hermeneutic circle. Initially, we read the transcribed interviews to familiarize ourselves with the data. We then generated initial codes to capture meaningful elements and organized these into overarching themes. After reviewing and refining the themes to ensure they accurately represented the data, we clearly defined and named each one. Finally, we provided detailed descriptions of each theme to articulate its significance and implications. Thus, thematic analysis was conducted within, and informed by, the hermeneutic framework rather than as a separate analytical procedure.

A condensed overview of codes and their linkage to subthemes and final themes is provided as Supporting File C.

The team consisted of female researchers with clinical experience in nursing and healthcare, as well as academic expertise in qualitative and quantitative methodologies. All members had previous experience with hermeneutic and thematic analysis. Throughout the analytical process, regular team discussions were conducted to reflect on pre‐understandings, professional assumptions, and interpretive positioning, ensuring reflexivity and analytical transparency, as seen in Table 1.

**TABLE 1:** Contains preconception notes for three researchers.

Researcher	Role/background	Expectations/assumptions	Possible biases	How to manage	Reflection prompt
Researcher 1—cross‐sectoral, interdisciplinary researcher with user involvement focus	Experience with cross‐sector projects and interdisciplinary collaboration; works with user involvement in service design	Meaningful involvement of patients/relatives will improve relevance and sustainability; barriers often stem from poor communication and unclear roles	Risk of over‐valuing participant perspective, and to assume cross‐disciplinary solutions are superior	Record memos capturing both user‐driven perspectives and system constraints	Which user perspective did I favor, and which system factors pointed to limitations?
Researcher 2—researcher in neurology	Clinician in a neurology dept; experience with stroke, dementia, rehab	Patients' accounts will focus on functional loss, cognitive/communication difficulties and rehab needs. Clinical priorities (safety, follow‐up) will weigh heavily	Risk to priorities clinical severity, focus on hospital‐based solutions	Write a memo each time a statement is interpreted as a “clinical problem”; seek alternative readings; foreground patient quotes	Which passages led me to prioritize a clinical explanation, and which quotes challenged that assumption?
Researcher 3—action researcher	Researcher experienced in action research and implementation; focus on change and iterative improvements	Data will point to concrete improvement opportunities; participants will be oriented toward solutions	Tendency to emphasize implementable findings and underplay deeper interpretative possibilities; risk of interpreting statements in light of changeability	Keep memos distinguishing descriptive findings from implementation suggestions; document negative cases showing why user involvement might fail	When has my action‐oriented stance shaped the selection of solutions, and what data point against these solutions?

### 1.6 Ethical Considerations

This study complied with the Act on Research Involving People and followed established ethical guidelines [33]. The regional Ethics Committee approved the study REG‐050‐2019. Informed consent was obtained from all participants before their involvement. This process included oral and written explanations of the study, ensuring that participants understood their rights to anonymity and their ability to withdraw without repercussions for their treatment or future healthcare needs. Anonymised data supporting the findings of this study are stored securely at Region Zealand. Due to ethical and legal restrictions under Danish data protection law, data cannot be made publicly available.

This approach aligns with the fundamental principles of respect for individual autonomy and the right to self‐determination as outlined in the Declaration of Helsinki [34].

## 2 Results

Participant quotations are presented to illustrate key themes and provide authentic insights into patients' and relatives' experiences. Each theme is subsequently interpreted considering the study's hermeneutic framework to clarify the underlying meaning and theoretical significance of participants' narratives.

A total of 19 interviews were conducted. Immediately after completing the V4M at the hospital, 11 patients and six of their relatives were interviewed. Five relatives were unable to participate in the hospital interviews. Additionally, 14 days after hospital discharge, seven patients and their relatives were interviewed. The ages of the patients ranged from 73 to 98 years, and the number of diagnoses per patient ranged from 7 to 14 (Table 2). Notably, three patients had passed away before the second interview. One of the patients also said that she did not remember much from the V4M

“No, I don't remember much—I was too sick to attend in those days. It was good that my daughter was with me”

(pt‐4, interview at home)

These findings indicate that we are dealing with an older adult group with multimorbidity requiring integrated care across various disciplines and sectors.

**TABLE 2:** Patients' and relatives' characteristics.

Patient	Gender female (F) male (M)	Age	Number of registered diagnosis	Lives arrangement and relationship	Patient/relatives interview hospital	Patient/relatives interview home	Comment
Pt‐1	F	87	7	Lives own house with husband	x/‐	x/R‐1, husband	
Pt‐2	F	83	12	Nursing home resident	x/‐		Deceased before second interview
Pt‐3	F	73	14	Lives in own house with wife	x/R‐3, wife	x/R‐3, wife	
Pt‐4	F	82	13	Nursing home resident	x/‐	x/R‐4, daughter	
Pt‐5	M	76	12	Lives alone in own house	x/R‐5, son		Too unwell to participate in follow‐up interview
Pt‐6	M	80	9	Lives in own house with wife	x/‐	x/R‐6, daughter	
Pt‐7	F	83	7	Lives alone in own house	x/R‐7, grandson	x/R‐7, grandson	
Pt‐8	M	82	8	Lives alone in own house	x/‐	x/R‐8, sister	
Pt‐9	M	92	11	Lives alone in own house with wife	x/R‐9, daughter	x/R‐9, daughter	
Pt‐10	M	98	8	Lives alone in own house	x/R‐10, son		Deceased before follow‐up interview
Pt‐11	F	74	7	Lives alone in own house	x/R‐11, daughter		Deceased before follow‐up interview

Abbreviations: F = female; M = male; Pt = patient; R= relatives.

### 2.1 Theme 1: Bridges Between System Patients' Experiences

Before participating in the V4M, patients described transitional care as fragmented, characterized by uncertainty, limited involvement, and unclear communication across sectors. Several patients felt caught between hospital and general practice, particularly regarding medication changes and treatment decisions. As one patient explained:

“Often, when I see my GP after a hospitalization, she asks why the hospital changed my medication. I don't know. It makes me feel unsafe. It's like they see my illness differently.”

(pt‐3, interview at home)

This experience illustrates how sectoral discontinuity placed responsibility for coherence on the patient, contributing to insecurity and a sense of marginalization. Participation in the V4M marked a clear shift in patients' experiences. Patients described how the meeting created a shared space where professionals communicated directly with one another and actively invited the patient's perspective. One patient stated:

“At the meeting, they talked to each other and asked me what I wanted.”

(pt‐11, interview at hospital)

Patients' priorities were not only voiced but also translated into concrete clinical actions. For example, one patient redirected attention to a chronic lung condition that had previously been overlooked:

“I told them about my lung disease. It was my biggest concern, even though I was hospitalized with heart problems. We discussed it at the meeting (V4M), and I was seen by a pulmonologist who changed my treatment. Now I don't need oxygen therapy anymore.”

(pt‐1, interview at home)

This demonstrates how the V4M enabled patients to influence care planning across diagnoses and sectors, thereby strengthening continuity and relevance of care. Patients also emphasized that meaningful involvement did not depend on avoiding medical terminology, but on being taken seriously and having plans explained in relation to their own needs:

“It didn't matter that they used medical language. Most importantly, they made a plan based on my needs and explained it to me.”

(pt‐9, interview at home)

Trust in the healthcare system was further strengthened when patients observed professionals sharing responsibility across sectors. One patient described how coordinated dialog during the V4M led to tangible changes in municipal care:

“For a long time, I had experienced that the help I received from the municipality was insufficient. […] After this meeting (V4M), inexperienced helpers no longer come to me.”

(pt‐3, interview at home)

#### 2.1.1 Relatives' Experiences

Relatives often described feeling excluded from discharge planning prior to the V4M, despite possessing essential knowledge about the patient's daily life and vulnerabilities. One daughter expressed concern that decisions had already been made without family input:

“We were nervous about the discharge because Dad was going home to an empty house. […] It was as if they had already decided that he was going home.”

(R‐6, interview at home)

After participating in the V4M, relatives more frequently experienced being acknowledged as partners in planning. Their involvement provided clarity about roles and expectations and reduced uncertainty regarding post‐discharge responsibilities. One relative described the relief this created:

“It was a great relief to plan my father's discharge together. I knew what was expected of me, and I wouldn't have felt that way without the meeting.”

(R‐10, interview at hospital)

#### 2.1.2 Interpretation

The theme *Bridges between Systems* illustrates how the V4M functioned as a turning point in patients' and relatives' experiences of transitional care. Prior to the intervention, participants described a fragmented system in which responsibility for coherence often fell on patients and families themselves. From the perspectives of patients and relatives, the V4M altered this dynamic by creating a shared, cross‐sectoral space where information, responsibility, and decision‐making were aligned. Patients' narratives indicate a shift from passive recipients of care to active contributors whose experiences and priorities actively informed dialog, influenced clinical reasoning, and shaped cross‐sectoral care planning. This contributed to restored trust and a sense of safety. For relatives, participation represented a movement from marginal observers to engaged collaborators, reducing feelings of exclusion and uncertainty. Patients and relatives accounts suggest that the V4M supported a form of relational professionalism in which professional authority was exercised through dialog rather than hierarchy, responsibility was coordinated across sectors rather than delegated to patients or relatives, and patients' and relatives' experiential knowledge was recognized as a legitimate and necessary contribution to transitional care. In this form of professionalism, clinical expertise and lived experience were brought into relation, enabling care decisions to be both medically sound and contextually meaningful.

### 2.2 Theme 2: A Relational Space of Alignment

#### 2.2.1 Patients' Experiences

Patients and relatives described the V4M as a turning point in the discharge process, and experienced it as a structured space in which fears could be expressed, needs acknowledged, and plans discussed collaboratively. Patients emphasized that what mattered most was not efficiency alone, but the experience of being seen, heard, and taken seriously. For some patients, this involved making ethically and emotionally complex treatment decisions. One patient described how her longstanding wishes to discontinue dialysis was recognized and supported during the V4M:

“I have told John (GP) several times that I do not want to be on dialysis. He supported that decision at this meeting (V4M). This meant that the doctor at the hospital changed my treatment. I was happy about that.”

(pt‐2, interview at hospital)

Prior to the V4M, patients often experienced fragmented care trajectories, where they were required to repeat their medical histories and restate their preferences to multiple professionals. The V4M altered this experience by creating a shared communicative platform. As one patient explained:

“It was reassuring when everyone had heard the same thing and knew what was going to happen, and I didn't have to repeat myself.”

(pt‐3, interview at home)

The meeting also enabled sensitive conversations about autonomy, vulnerability, and dependence. One patient described the tension between her wish to remain independent and her family's concerns:

“I'm afraid I won't be able to climb the stairs to my house when I get home. Accepting more help is hard; I want to care for myself. Chris (grandson) is also worried about me. He wants me in a nursing home. But I don't want that. They said that together with the municipality's nurse and Harry (GP), we must find a solution so that I can stay in the house. They respected my wish.”

(pt‐7, interview at hospital)

The account illustrates how fears about functional decline could be expressed without the patient experiencing a loss of autonomy.

Patients described how hearing a unified plan across sectors helped them gradually accept their care needs and reduced uncertainty about the future:

“They said we'd find a solution with the municipal nurse.” (pt‐8, interview at hospital). And “It was important to me that the GP informed me about my medical history, that everyone heard the same thing and could work according to the same plan.”

(pt‐1, interview at home)

#### 2.2.2 Relatives' Experiences

Relatives frequently described the emotional and practical strain of coordinating care and acting as intermediaries between sectors. The V4M provided a setting where this burden could be articulated and, to some extent, shared. However, the meeting also exposed emotional vulnerabilities. One daughter recalled how her father became distressed when new medical information was introduced:

“My father was upset because he didn't know that he also had kidney disease. The doctors discussed the treatment extensively, which my father found challenging to comprehend.”

(R‐9, interview at home)

Despite such moments, relatives emphasized the value of the clarity and recognition that the V4M offered. One relative described how the meeting made her own limits visible and legitimate:

“At this meeting (V4M), I realized I could no longer do it alone. There was great sensitivity to my assessment of the help Maria and I require.”

(R‐1, interview at home)

These accounts highlight both the emotional complexity of family caregiving and the relief that can arise when responsibility is acknowledged and shared.

#### 2.2.3 Interpretation

The theme *A Relational Space of Alignment* illustrates how the V4M functioned as a structured forum in which emotional, relational, and practical dimensions of transitional care unfolded simultaneously. The findings show that patients valued not only coordinated planning, but the quality of interaction—being listened to, recognized, and included in decisions that affected their lives.

The V4M enabled vulnerability to coexist with autonomy by providing a structured and relational setting in which patients could express fear, uncertainty, and dependence without relinquishing control over decisions that mattered to them. By bringing professionals and relatives into the same conversation, patient concerns could be acknowledged without undermining self‐determination. This points to a form of relational autonomy, where decisions were negotiated between the patient's wishes, family concerns, and the healthcare system's possibilities. Relatives' reflections further demonstrate how the V4M made previously invisible caregiving work visible and legitimate. The structured meeting allowed family members to articulate both their commitment and their limits, thereby opening space for shared responsibility. From the perspectives of patients and relatives, the V4M appeared to involve healthcare professionals coordinating across sectors in ways that were experienced as empathetic, attentive, and sensitive to the emotional consequences of information sharing. Overall, this theme shows how the V4M transformed the discharge conversation into a space of alignment, bringing together multiple perspectives to foster mutual understanding, emotional safety, and trust.

### 2.3 Theme 3: Involvement and Responsibility Are Deeply Interconnected

#### 2.3.1 Patients' Experiences

Before participating in the V4M, several patients described feeling uncertain and carrying a disproportionate responsibility for coordinating their own care transitions. They lacked clarity regarding follow‐up care, ongoing treatment, and the type of support available after discharge. This uncertainty contributed to anxiety and a sense of being left alone with complex care decisions. After participating in the V4M, patients consistently reported that active involvement in discharge planning fostered feelings of safety and trust. Being invited to express what mattered most appeared, from the patients' perspectives, to shift responsibility back to the healthcare system by making professional accountability visible and explicit—patients no longer felt that they alone were responsible for ensuring follow‐up, coordination, and continuity of care after discharge. One patient explained:

“When I say what matters most to me, I feel like they take responsibility for what matters.”

(pt‐11, interview at hospital)

Patients also emphasized the importance of having all relevant professionals present at the same meeting. Knowing that responsibilities were discussed openly and allocated reduced both emotional and practical uncertainty:

“When all professionals attend and are assigned responsibilities, it minimizes anxiety.”

(pt‐3, interview at home)

Together, these accounts illustrate how being heard and having clear agreements in place helped patients feel protected during the vulnerable transition from hospital to home.

#### 2.3.2 Relatives' Experiences

Relatives frequently described acting as informal care coordinators, transferring information between hospital staff, GPs, and municipal services. This intermediary role often resulted in stress, exhaustion, and a persistent sense of responsibility for ensuring continuity of care. Participation in the V4M was experienced as a significant relief, as responsibility was perceived to be redistributed back to the healthcare system. One relative described this shift as follows:

“It is a great relief for me that I do not have to be a mediator between all the agencies, but that they talk to each other and agree on who is responsible for what.”

(pt‐9, interview at home)

Several relatives also emphasized that being included in the discussion strengthened their trust in the discharge process and reduced the burden of acting as a communication link:

“It was a relief to know that all healthcare professionals were aware of my dad's illness, situation, and plan… so that we didn't have to act as a link between the treating doctor at the hospital and the GP.”

(pt‐6, interview at home)

These accounts show how relatives experienced the V4M as a protective framework that clarified roles and responsibilities and reduced the mental load associated with fragmented communication.

#### 2.3.3 Interpretation

The theme *Involvement and Responsibility Are Deeply Interconnected* illustrates how patients and relatives linked meaningful involvement with shared accountability. When patients' voices were heard and reflected in concrete plans during the V4M, they experienced reassurance that responsibility for follow‐up care had been assumed by the system.

From the perspectives of patients and relatives, the V4M provided a transparent structure in which responsibilities were discussed and clarified. This visibility replaced diffuse and implicit accountability with clearer expectations about who would do what after discharge. As a result, involvement was experienced not as a symbolic gesture, but as a tangible sense of reliability and follow‐through. For relatives, this redistribution of responsibility represented emotional relief and validation. Their experiential knowledge and practical constraints were acknowledged as relevant to planning, positioning them as active contributors rather than peripheral supporters. Overall, this theme underscores that user involvement in transitional care was experienced not only as having a voice but also as being protected by a system willing to listen, respond, and assume shared responsibility for agreed‐upon actions.

## 3 Discussion

This qualitative study explored how older adults with multimorbidity and their relatives experienced user involvement in *transitional care* through V4M. The findings demonstrate that V4M provided a structured, relational, and cross‐sectoral framework that strengthened patient and family participation in discharge planning. The discussion below integrates these findings with existing literature to highlight how V4M promoted continuity, emotional safety, and shared accountability in transitional care.

The theme *Bridges between Systems* illustrates how V4M reduced fragmentation and uncertainty by enabling professionals across hospitals, municipalities, and GPs to communicate in real time. This finding aligns with previous research emphasizing that disjointed communication and unclear role distribution increase insecurity among older patients and their families [35]. Similar to van Grootel et al. [18] and Birkeland et al. [15], our results show that when the patient's voice becomes central to dialog, coordination improves, and care plans more accurately reflect patients' priorities. Through V4M, participants perceived that responsibility was redistributed—from individuals navigating the system alone to a shared professional network supporting them. This confirms the value of structured, intersectoral models that promote continuity and patient trust [19, 36].

The theme *A Relational Space of Alignment* highlights that involvement extends beyond information exchange—it depends on emotional recognition and mutual understanding. Patients valued being listened to and respected, even when they did not participate in clinical decision‐making. This finding supports Vrangbæk [36, 37] and McColl‐Kennedy et al. [38], who argue that genuine user involvement requires emotional engagement and the acknowledgment of patients' experiences. Our results suggest that the relational quality of communication—rather than its procedural form—was decisive for patients' sense of inclusion [36, 37]. While some participants emphasized that feeling acknowledged and having their concerns translated into a concrete plan mattered more than avoiding medical terminology, this should not be interpreted as medical language being unproblematic. Instead, our findings indicate that professional use of medical language requires relational and communicative competence, including sensitivity to patients' understanding and opportunities for clarification; without such efforts, medical jargon may constitute a barrier to genuine collaboration. V4M created a safe space where patients could express vulnerability without losing autonomy, consistent with concepts of relational autonomy and relational professionalism [18, 39].

For relatives, this relational alignment also reduced emotional strain. The V4M validated their knowledge and caregiving efforts, turning them from peripheral supporters into acknowledged co‐participants. This is consistent with earlier findings that caregivers' involvement enhances discharge quality and reduces the risk of readmission [40, 41]. In contrast to prior work showing that relatives often feel excluded or overburdened [42], our study found that structured inclusion during V4M created a sense of recognition and emotional relief.

The theme of Involvement and Responsibility, which are deeply interconnected, reveals that participants equated genuine involvement with the system's willingness to assume and share responsibility. In this study, *responsibility* refers to professional and organizational accountability for coordinating care, ensuring follow‐up, and translating patients' expressed priorities into concrete actions across sectors. It does not refer to responsibility placed on patients or relatives, but rather to how involvement was experienced as meaningful when healthcare professionals visibly assumed and shared responsibility for agreed plans. Patients expressed trust when their concerns were acknowledged and translated into concrete follow‐up actions. This reciprocal exchange between expression and response resonates with the principles of co‐production, where involvement generates shared accountability [22, 43]. By defining roles and tasks explicitly, V4M turned participation into an actionable practice rather than a symbolic consultation. Our findings suggest that it is not video technology per se that fosters involvement, but the deliberate orchestration of relational, temporal, and accountability structures within the V4M format.

Relatives described relief when coordination duties shifted from them to professionals, mirroring findings from studies on caregiver burden during discharge [44, 45]. This redistribution of responsibility transformed the discharge process from a source of anxiety into a collaborative partnership. It further supports the argument that user involvement must be operationalized through visible, accountable structures rather than aspirational ideals.

### 3.1 Implications for Practice

Taken together, the three themes illustrate that meaningful involvement in transitional care requires both structural coordination and relational competence. V4M offered a mechanism for bridging these dimensions. The intervention not only facilitated communication but fostered shared understanding and trust among sectors. This dual impact is consistent with recent calls for person‐centered, cross‐sectoral approaches that combine dialog, transparency, and accountability [35, 40]. Healthcare professionals should be trained not only in coordination tools but also in communicative and relational skills that support empathy and inclusion. Integrating structured meetings, such as V4M, into discharge workflows could enhance continuity and reduce the risk of fragmented responsibilities. However, emotional readiness and time constraints among professionals remain challenges to be addressed during implementation.

### 3.2 Strengths and Limitations

This study provides several strengths. First, it provides new insights into how user involvement is experienced in V4M, a relatively underexplored model for transition care. The hermeneutic qualitative approach enabled an in‐depth understanding of patients' and relatives' experiences. Conducting two rounds of interviews allowed the research team to capture both immediate reactions and more reflective perspectives after discharge. Involving patients and their relatives provides a more holistic understanding of the transition care process, especially for older adults with multimorbidity. However, several limitations need to be acknowledged. The study was conducted in a single regional context in Denmark, which may limit the transferability of findings to other health systems with different structures or cultures. Participants in V4M, and in the interviews, were selected based on their cognitive and physical ability to engage in a video‐based meeting and follow‐up interviews, potentially excluding more fragile or marginalized individuals. These groups are common in transitional care contexts, and their exclusion may limit the transferability of the findings. The results therefore primarily reflect the experiences of older adults who were cognitively able and sufficiently stable to participate in a video‐based discharge planning meeting. In addition, the study focused exclusively on patients' and relatives' experiences; the perspectives of healthcare professionals involved in V4M were not captured, which could have enriched the interpretation of cross‐sectoral collaboration. Future research can address these limitations by including a broader participant base and by triangulating user perspectives with those of healthcare professionals involved in care transitions.

## 4 Conclusion and Future Research

This study explored how older adults with multimorbidity and their relatives experienced user involvement in transitional care through the V4M approach. The findings demonstrate that user involvement was experienced as meaningful when it was embedded in a structured, cross‐sectoral dialog that combined relational engagement with clear accountability. Rather than involvement being perceived merely as having a voice, participants associated involvement with feeling protected by a system that listened, responded, and assumed responsibility for agreed actions. Across the three themes, V4M functioned as a mechanism for reducing fragmentation by bringing relevant professionals, patients, and relatives into a shared communicative space before discharge. This alignment enabled patients' priorities and experiential knowledge to inform care planning and supported relatives in articulating both their commitment and their limits. In this way, V4M shifted responsibility away from patients and families acting as informal coordinators and back toward the healthcare system as a collective actor. The study contributes to existing literature by demonstrating that user involvement in transitional care is inseparable from visible and shared responsibility. Involvement was experienced as reassuring when patients and relatives could see how professional accountability was coordinated across sectors. This highlights that relational aspects of care—such as recognition, emotional safety, and trust—must be supported by concrete organizational structures if involvement is to move beyond symbolic participation.

While V4M appears to offer a promising model for person‐centered and integrated transitional care, the findings also indicate that meaningful involvement depends on patients' capacity to participate and on professionals' relational and communicative competencies, particularly when complex or sensitive information is discussed. Relatives played a crucial role in bridging understanding and ensuring feasibility of discharge plans, underscoring the importance of their structured inclusion.

Future research should examine how V4M can be adapted to include more frail or cognitively vulnerable patients and explore healthcare professionals' perspectives on responsibility‐sharing in cross‐sectoral settings. Quantitative and mixed‐methods studies are also needed to assess the impact of V4M on clinical outcomes, resource use, and scalability across different healthcare systems.

## Author Contributions

**Ditte Høgsgaard:** conceptualization, investigation, funding acquisition, writing – original draft, methodology, validation, writing – review and editing, formal analysis, project administration. **Janet Froulund Jensen:** investigation, writing – original draft, methodology, writing – review and editing, resources.

## Conflicts of Interest

The authors declare no conflicts of interest.

## Supporting information

SupplementA.intereviewGuide.

SUPPL.B.COREQ.

SupplementC.

## Data Availability

Data sharing is not applicable to this article, as no new data were created or analyzed in this study.
