# Supplementary material for: User Involvement in Transition Care in Virtual 4‐Party Meetings: A Qualitative Study
Source: Health Expect. 2026 Jan 24;29(1):e70566. doi: 10.1111/hex.70566 (PMC12831168; doi:10.1111/hex.70566)
Supplement: Supplementary file 1 — SupplementA.intereviewGuide. [file HEX-29-e70566-s003.docx]

# SUPPLEMENT A: Interview Guides for V4M Study

**Guide 1: Immediate post-V4M interview**

**Aim**: Capture immediate experiences, understanding of roles/responsibilities, reactions to be involved in the planning of discharge.
Suggested duration: 20–35 minutes.

### Opening / Warm-up (2–4 min)

- Thank participant and repeat purpose: 'Thank you for wanting to talk to me. I would like to hear about your experience of today's meeting.'
- Confirm it's a good time to talk.

### Main questions

1. How did you experience the meeting today (V4M)?

What stood out most? How was the atmosphere? Did you feel safe?

What happened in the meeting that was important to you?

How were your responsibilities/others' responsibilities presented? Was it clear who would do what?

Who took responsibility for medication, home help, follow-up? Were you in agreement with the distribution?

1. Did you feel heard and involved in the decisions?

Did you give input? How did professionals respond to your wishes?

1. What are your feelings about the next steps/discharge?

What worries you most right now? What do you feel confident about?

1. Was there anything you did not understand or would like to have elaborated?

Language, jargon, clarity of action plan.

5. To relatives: How did you experience being included? Did you receive relevant information and support?

### Closing (2–3 min)

- Anything you would like to add that I haven't asked?
- Inform about the next interview (approx. 14 days) and thank participant.

## Guide 2. 14 days after discharge (Follow-up)

**Aim**: Capture reflective perspectives on how agreements were realized, experienced barriers/changes, patient agency, and effects on daily life.
Suggested duration: 25–45 minutes.

### Opening / Warm-up (2–4 min)

Confirm identity and reference to previous meeting: 'You participated in V4M on [date]. I would like to hear how it has been since then.'

### Main questions

1. How did the transition home go?

Was help provided on time? Were the instructions clear? What went well / poorly?

2. Were the agreed actions from V4M followed up in practice?

Who did what? Were some agreements not implemented? What were the reasons?

3. How do you experience your involvement, role now in relation to follow-up/decisions?

Do you feel more active/secure or left on your own?

4. Have your relationships with professionals changed since the meeting?

Do you feel trust? Is there better continuity or contact points?

5. What concrete consequences has V4M had for your daily life (practical, psychological)?

Medication changes, assistive devices, cleaning, anxiety, sleep, mobility.

6. If you could change one thing about the meeting/transition, what would it be?

More time, clearer written info, a different form of involving relatives?

7. To relatives: How did you experience being included? Did you receive relevant information and support?

### Closing (2–3 min)

Summarise key points. Request permission to quote anonymised excerpts. Thank participant and remind about data storage.
