# Supplementary material for: User Involvement in Transition Care in Virtual 4‐Party Meetings: A Qualitative Study
Source: Health Expect. 2026 Jan 24;29(1):e70566. doi: 10.1111/hex.70566 (PMC12831168; doi:10.1111/hex.70566)
Supplement: Supplementary file 2 — SUPPL.B.COREQ. [file HEX-29-e70566-s002.docx]

# SUPPLEMENT B

# COREQ: Consolidated Criteria for Reporting Qualitative Research – 32-item Checklist

## Domain 1: Research team and reflexivity

| Item | Description / Page reference |
| --- | --- |
| 1. Interviewer/facilitator | All interviews were conducted by the first author to ensure consistency. (Methods, section 3.4 Data collection). |
| 2. Credentials | First author and Co-authors are Nurses and researchers with experience in qualitative and quantitative studies. (Acknowledged in section 3.5 and researcher background description.) |
| 3. Occupation | At the time of the study, the first author was a researcher affiliated with a university and a hospital. |
| 4. Gender | The interviewer was female. (Methods, 3.4.) |
| 5. Experience and training | The interviewer had extensive experience in qualitative interviewing and hermeneutic analysis. (Methods, 3.5.) |
| 6. Relationship established | No prior relationship with participants was established before study commencement. (Methods, 3.3 Participants.) |
| 7. Participant knowledge of the interviewer | Participants received written and oral information about the study purpose and researcher roles via the ethics-approved consent process. (Methods, 3.6 Ethical considerations.) |
| 8. Interviewer characteristics | The interviewer’s interest in user involvement and cross-sectoral care was stated as part of the research aim. Reflexivity was ensured through group discussions of pre-understandings before coding. (Methods, 3.5 Data analysis.) |

## Domain 2: Study design

| Item | Description / Page reference |
| --- | --- |
| 9. Methodological orientation and theory | Hermeneutic approach based on Gadamer’s philosophical hermeneutics combined with reflexive thematic analysis (Braun & Clarke). (Methods, 3.1 & 3.5.) |
| 10. Sampling | Purposive sampling of patients +65 years with multimorbidity who participated in V4M. Relatives included if identified by patients. (Methods, 3.3 Participants.) |
| 11. Method of approach | Participants were recruited face-to-face during hospitalization through the V4M program and invited to participate in interviews. (Methods, 3.3.) |
| 12. Sample size | N = 19 interview sessions (11 patients, 6 relatives in round 1; 7 patient-relative pairs in round 2). (Results, section 4.) |
| 13. Non-participation | Two patients died before the completion of second interview. One patient was too ill to participate in the second interview. Some relatives were unable to participate in the first interview due to scheduling or health constraints. (Results, section 4.) |
| 14. Setting of data collection | Interviews were conducted at the hospital immediately after V4M or at participants’ homes/nursing home 14 days post-discharge. (Methods, 3.4.) |
| 15. Presence of non-participants | Only participants and the interviewer were present. |
| 16. Description of sample | Patients aged 73–98 years with 7–14 chronic conditions. (Results, Table 1.) |
| 17. Interview guide | A semi-structured interview guide was developed, focusing on experiences of involvement and collaboration. (Methods, 3.4.) |
| 18. Repeat interviews | Yes – two interview rounds (immediately after V4M and 14 days post-discharge) to capture immediate and reflective experiences. (Methods, 3.4.) |
| 19. Audio/visual recording | Interviews were audio-recorded and transcribed verbatim. (Methods, 3.4.) |
| 20. Field notes | Field notes were made after interviews to document impressions and context. (Methods, 3.4.) |
| 21. Duration | Interviews lasted 15–40 minutes (mean 28). (Methods, 3.4.) |
| 22. Data saturation | Saturation is discus in Data collection (section 3.4.) |
| 23. Transcripts returned | Transcripts were not returned; however, interpretation was validated through iterative group reflection and theme discussions. (Methods, 3.5.) |

## Domain 3: Analysis and findings

| Item | Description / Page reference |
| --- | --- |
| 24. Number of data coders | Three researchers participated in coding and theme validation. (Methods, 3.5.) |
| 25. Description of the coding tree | Themes were developed inductively through iterative movement between parts and whole following Gadamer’s hermeneutic circle and Braun & Clarke’s six-step model. (Methods, 3.5.) |
| 26. Derivation of themes | Themes were derived from the data. (Methods, 3.5 and Results, section 4.) |
| 27. Software | Data were manually coded without qualitative software. (Methods, 3.5.) |
| 28. Participant checking | Participants did not review findings, but validation occurred via reflexive dialogue in the research team. (Methods, 3.5.) |
| 29. Quotations presented | Participant quotations are presented to illustrate each theme, identified by participant number and interview context (e.g., pt-3, at home). (Results, section 4.) |
| 30. Data and findings consistent | Yes – quotations clearly support the thematic interpretations. (Results, Themes 1–3.) |
| 31. Clarity of major themes | Three major themes were clearly defined and presented: Bridges between systems; A relational space of alignment; Involvement and responsibility are interconnected. (Results, section 4.) |
| 32. Clarity of minor themes | Subthemes and nuanced cases were described to show variation in experiences among patients and relatives. (Results, section 4.) |
