# Supplementary material for: User Involvement in Transition Care in Virtual 4‐Party Meetings: A Qualitative Study
Source: Health Expect. 2026 Jan 24;29(1):e70566. doi: 10.1111/hex.70566 (PMC12831168; doi:10.1111/hex.70566)
Supplement: Supplementary file 3 — SupplementC. [file HEX-29-e70566-s001.docx]

# SUPPLEMENTAL FILE C Consolidated Analytic Table — Themes 1–3

| Theme | Subtheme | Brief description | Key codes (condensed) | Illustrative quote (short) | Analytic / reflexive anchor |
| --- | --- | --- | --- | --- | --- |
| Theme 1: Bridges between systems | Relational continuity | Trust and safety emerge when professionals know the patient across sectors and act on shared history. | continuity_of_care; shared_history | “Often, when I see my GP after a hospitalization, she asks why the hospital changed my medication. I don’t know. It makes me feel unsafe.” — pt-3 | Continuity interpreted as relational safety rather than only organisational structure; supported by negative case analysis. |
|  | Shared responsibility & coordination | V4M clarifies roles and distributes responsibility across hospital, GP, and municipality. | role_clarity; shared_responsibility | “Each meeting ends with a written summary that confirms responsibilities across sectors.” | Reflexive shift from fragmentation-as-default to structure-as-relief documented in analytic memos. |
|  | Amplified patient voice | Patients move from passive recipients to active contributors influencing care decisions. | patient_agency; shared_decision | “At the meeting, they talked to each other and asked me what I wanted.” — pt-11 | Hermeneutic interpretation of agency as dialogical rather than individual. |
|  | Inclusion of relatives | Relatives are recognised as knowledgeable partners rather than peripheral observers. | relative_inclusion; caregiver_knowledge | “We were nervous about the discharge… It was as if they had already decided.” — R-6 | Reflexive awareness of relatives’ invisible coordination work shaped theme development. |
|  | Institutional responsiveness | Trust may derive from system responsiveness rather than personal continuity alone. | system_responsiveness | “After this meeting, inexperienced helpers no longer come to me.” — pt-3 | Negative cases nuanced assumption that continuity is the sole route to trust. |
| Theme 2:  A relational space of alignment | Shared narrative | All actors hear the same story, reducing repetition and misalignment. | shared_narrative; alignment | “It's reassuring when everyone has heard the same thing.” — pt-3 | Shift from information-transfer logic to dialogical meaning-making. |
|  | Emotional safety | V4M enables expression of fears and vulnerabilities without loss of dignity. | emotional_safety; recognition | “They respected my wish.” — pt-7 | Clinical preconceptions of safety reframed toward emotional reassurance. |
|  | Relational autonomy | Autonomy negotiated through dialogue rather than binary decisions. | relational_autonomy | “They said we’d find a solution with the municipal nurse.” — pt-8 | Autonomy interpreted as relationally constituted. |
|  | Professional alignment | Cross-sector presence legitimizes decisions and fosters coherence. | professional_alignment | “Everyone heard the same thing.” — pt-1 | GP role emerged as credibility anchor through cross-case comparison. |
|  | Managing difficult knowledge | Shared spaces may surface sensitive diagnoses requiring careful facilitation. | difficult_information | “My father was upset because he didn’t know he had kidney disease.” —-9 | Negative case challenged assumption that more transparency is always beneficial. |
| Theme 3: Involvement and responsibility are deeply interconnected | Involvement as responsibility transfer | Being heard signals that responsibility is assumed by the system. | system_accountability | “When I say what matters most to me, I feel like they take responsibility.” — pt-11 | Involvement interpreted as moral and relational, not merely procedural. |
|  | Role clarity | Explicit task allocation reduces anxiety and uncertainty. | role_clarity | “When all professionals attend and are assigned responsibilities, it minimizes anxiety.” — pt-3 | Structural clarity shown to have emotional significance. |
|  | Relief for relatives | V4M relieves relatives of informal coordination burdens. | caregiver_relief | “I do not have to be a mediator between all the agencies.” — pt-9 | Reflexive shift from ‘relative as resource’ to ‘system responsibility’. |
|  | Trust through collaboration | Visible professional collaboration builds trust in follow-up care. | trust_in_system | “It was a relief to know that all professionals were aware of the plan.” — pt-6 | Trust linked to visibility of teamwork rather than promises alone. |
|  | Moral professional responsibility | Responsibility experienced as ethical commitment, not only task completion. | moral_responsibility | “They agree on who is responsible for what.” — R-9 | Clinical pre-understandings expanded toward relational ethics. |
